# 3D Printed Silicone Meniscus Implants: Influence of the 3D Printing Process on Properties of Silicone Implants

**DOI:** 10.3390/polym12092136

**Published:** 2020-09-18

**Authors:** Eric Luis, Houwen Matthew Pan, Anil Kumar Bastola, Ram Bajpai, Swee Leong Sing, Juha Song, Wai Yee Yeong

**Affiliations:** 1 Singapore Centre for 3D Printing, School of Mechanical and Aerospace Engineering, Nanyang Technological University, 50 Nanyang Drive, Singapore 639798, Singapore; g001@e.ntu.edu.sg (E.L.); ANILKUMA001@e.ntu.edu.sg (A.K.B.); slsing@ntu.edu.sg (S.L.S.); 2 School of Chemical and Biomedical Engineering, Nanyang Technological University, 70 Nanyang Drive, Singapore 639798, Singapore; matthew.pan@u.nus.edu (H.M.P.); songjuha@ntu.edu.sg (J.S.); 3 Center for Population Health Sciences, Lee Kong Chien School of Medicine, Nanyang Technological University, 11 Mandalay Road, Singapore 308232, Singapore; r.bajpai@keele.ac.uk

**Keywords:** additive manufacturing, 3D printing, silicone, meniscus implants, validation

## Abstract

Osteoarthritis of the knee with meniscal pathologies is a severe meniscal pathology suffered by the aging population worldwide. However, conventional meniscal substitutes are not 3D-printable and lack the customizability of 3D printed implants and are not mechanically robust enough for human implantation. Similarly, 3D printed hydrogel scaffolds suffer from drawbacks of being mechanically weak and as a result patients are unable to execute immediate post-surgical weight-bearing ambulation and rehabilitation. To solve this problem, we have developed a 3D silicone meniscus implant which is (1) cytocompatible, (2) resistant to cyclic loading and mechanically similar to native meniscus, and (3) directly 3D printable. The main focus of this study is to determine whether the purity, composition, structure, dimensions and mechanical properties of silicone implants are affected by the use of a custom-made in-house 3D-printer. We have used the phosphate buffer saline (PBS) absorption test, Fourier transform infrared (FTIR) spectroscopy, surface profilometry, thermo-gravimetric analysis (TGA), X-ray photoelectron spectroscopy (XPS), differential scanning calorimetry (DSC), and scanning electron microscopy (SEM) to effectively assess and compare material properties between molded and 3D printed silicone samples.

## 1. Introduction

Ten percent of the entire global population are elderly over the age of 65 suffering from osteoarthritis of the knee with meniscal pathologies [1,2]. More than one million cases of knee surgeries are performed annually in the US alone. However, current total meniscal substitute like NUSurface meniscal implant [3], which are currently undergoing Food and Drug Administration (FDA) Phase 2 Sun Clinical Trials, are not 3D printed and are not customizable. CMI [4] and Actifit [5,6] are the two natural and synthetic porous meniscal implants, respectively, used for symptomatic post-meniscectomy patients, provided that there are residual peripheral meniscus and minimal cartilage damage. They also suffer from the drawback of being weak mechanically and are approved for used in chronic meniscal injuries only.

The utilization of additive manufacturing technologies to develop highly customizable and application-oriented prototypes or mechanical parts have gained popularity in the past two decades [7]. More recently, even functional prototypes and end products have been made printable as the cost of 3D printers and inks became more affordable and more sophisticated softwares were developed [8]. However, current 3D printed scaffolds, using polyvinyl alcohol (PVA) or hydrogels [8,9,10], are mechanically weak and as a result patients are not able to execute immediate post-surgical weight-bearing ambulation and rehabilitation.

**Figure 1:**
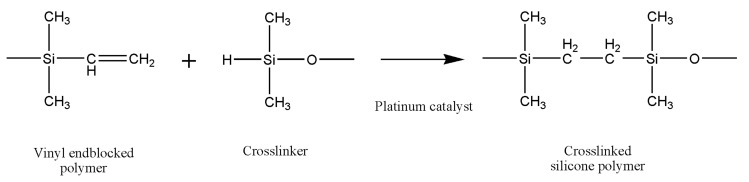
The hydrosilylation reaction of liquid silicone rubbers.

Liquid silicone rubbers (LSR) are silicone rubbers with additional vulcanization, and widely used in medical, construction, automotive, electronics, and the food industry, due to their excellent thermal and biocompatibility properties, low viscosity, controllable crosslinking and easy implementation. LSR are cross-linked by a hydrosilylation mechanism, which refers to the addition of Si–H bonds to carbon double bonds borne by poly(dimethylsiloxane) chains. LSR formulations are mainly composed of unsaturated silicone rubber (component A), polysiloxane has silane (Si–H) functional groups (component B) and platinum compound catalyst such as Karstedt’s catalyst [11]. The hydrosilylation reaction of LSR is presented in Figure 1.

A number of different types of 3D printing techniques have been used to 3D print the silicone elastomers and their composites, such as direct ink writing (DIW) 3D printing [12,13,14,15,16,17], digital light synthesis (DLS) 3D printing [18,19], inkjet 3D printing [20], drop-on-demand [21,22] and even modified fused deposition modeling (FDM) 3D printing [7,23,24]. The silicone elastomers and their composites are potential candidate materials in a number of different applications including soft sensors, actuators, and vibration control systems, see Bastola and Hossain [25] for a thorough review. However, the existing silicone elastomers or their composites are not biocompatible and are not suitable for meniscal implants. Thus, a bio-compactible and 3D printable silicone-based elastomer is always worthy of investigation.

The soft silicone-based elastomers, Ecoflex, and their composites are attractive in a number of applications such as skin-mountable strain sensors, wearable energy devices, and displays [26,27,28,29], by virtue of their mechanical properties. The excellent mechanical properties of Ecoflex make it a promising candidate material for human implants. However, biocompatible Ecoflex elastomer 3D printing for human implants has not been reported.

Combining the superior properties of silicone with the unmet needs of the meniscus market, we have developed a 3D silicone meniscus implant which encompasses the features of an ideal meniscal implant: (1) cytocompatible, (2) resistant to cyclic loading and mechanically similar to native meniscus, and (3) directly 3D printable. In our previous investigations [30,31], we presented the development of the in-house 3D printer, rheological properties of printing ink, and optimization of the printing process. The main focus of this study is to determine whether the purity, composition, structure, dimensions and mechanical properties of silicone implants are affected by the use of a custom-made in-house 3D printer based on an open source software and commercially available 3D printer.

In this study, we have used a phosphate buffer saline (PBS) absorption test, surface profilometry, X-ray photoelectron spectroscopy (XPS), Fourier transform infrared (FTIR) spectroscopy, thermo-gravimetric analysis (TGA), scanning electron microscopy (SEM), and differential scanning calorimetry (DSC) to effectively assess and compare material properties between molded and 3D printed silicone samples. SEM is used to assess the surface morphology of silicone samples and to investigate the relative changes in surface roughness [32]. FTIR is used for detection of chemical changes and surface hydroxylation in damaged samples. XPS is used to analyze elemental composition changes on the surface of the sample. TGA and DSC are used to analyze the thermal stability of silicone samples. American Society for Testing and Materials (ASTM) 575 compression testings [33] and cyclic loading up to 1000 cycles [34] are used to assess the mechanical stability and robustness of the silicone meniscus implants. The PBS absorption test [35], together with surface roughness measurements [36] is used to evaluate the degree of hydrophobicity of silicone samples [37]. The former measures the swelling content of the silicone sample, while the latter is a line-profiling method which produces a quantitative profile of the surface by using a high-resolution probe to detect changes in the surface topography. The degree of hydrophobicity is correlated to the affinity for attachment of the extracellular matrix and subsequent cell adhesion, proliferation and differentiation [33,38]. Despite using biocompatible silicone resin components A and B, the final 3D printed silicone implant should still be tested for biocompatibility. Cell viability and proliferation assays [39] with L929 fibroblasts are used to assess the cytocompatibility of silicone meniscus implants.

## 2. Materials and Methods

### 2.1. Experimental Set-Up

**Figure 2:**
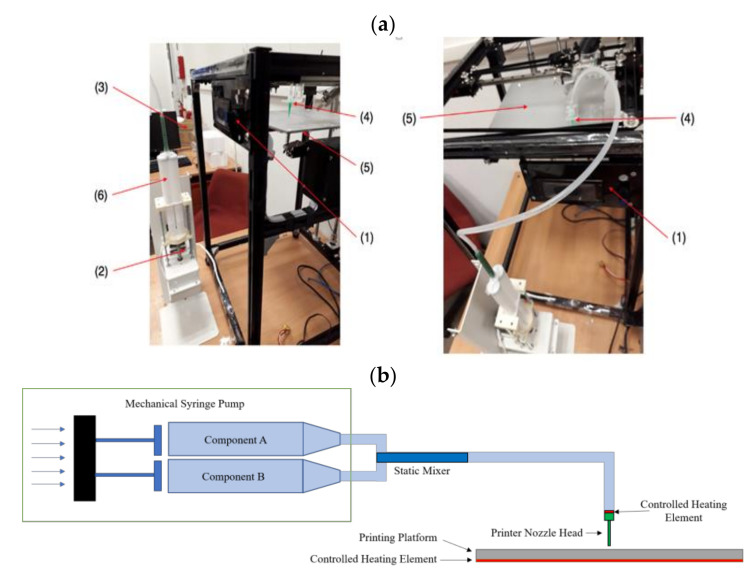
(**a**) Photographs: (1) motion control platform, (2) discovery extruder, (3) static mixer, (4) printer nozzle head, (5) heated printer bed platform, (6) double-barrel-syringe and (**b**) simplified schematic of experimental setup for heat-cure extrusion-based printer.

**Figure 3:**
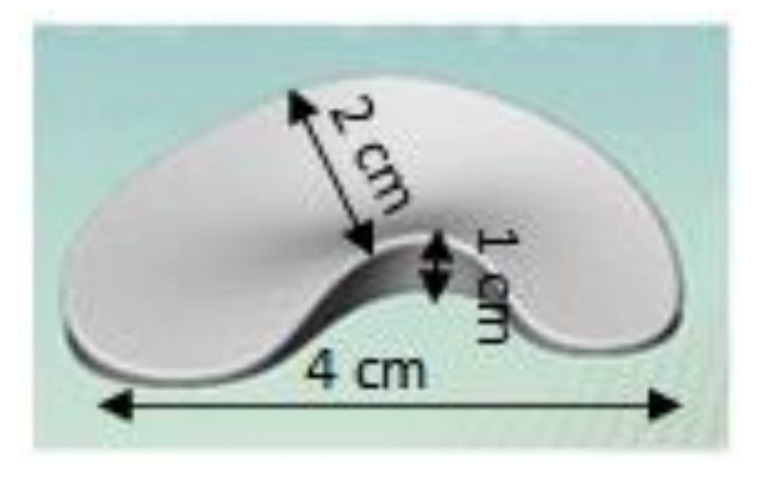
Computer-aided design (CAD) of meniscus implant.

The in-house silicone 3D extrusion printer is set up as shown in the diagram and schematic drawings in Figure 2. The heated platform has a printing area of 25 cm × 25 cm. A mechanical extrusion pump Model LSP02-1B (Longer Precision Pump Co., Ltd., Hebei, China) is set to an extrusion rate of 0.5 mL/min and connected to a 21G diameter heated nozzle via a 40 cm polyurethane tubing. The nozzle–platform distance is set at 15 mm. The print speed is set at 4800 mm/min. The stl file of the meniscus with dimension of (length) 4 cm × (width) 2 cm × (height) 1 cm is shown in Figure 3. The slic3r software (open source software) generates the G-code and determines the amount silicone deposited and subsequently the dimensional variations in extrusion-based 3D printing.

### 2.2. Sample Data and Dimensions

Commercially available LSR Ecoflex50 and Ecoflex 30 from Smooth-On Inc. (Macungie, PA, USA) are used in experiments. The sample technical data are given in Table 1. For compression tests, both standard cylindrical and 3D printed meniscus samples were used. The design of the specimen mold was based on ASTM D575 [33], consisting of a top plate with six cylindrical holes and a rectangular bottom part. The silicone elastomer does not adhere to the plastic mold during the curing process.

### 2.3. Surface Characterization

#### 2.3.1. Light Microscopy

Light microscopy using a Zeiss Axioskop Mat microscope (Carl Zeiss, Jena, Germany) with Olympus ACH 1× lens at 20× magnification is used to first assess the general surface morphology, discoloration and any surface tears or defects of the silicone implants.

#### 2.3.2. Scanning Electron Microscope

An SEM is then used to assess the phase morphology, surface topography and relative changes of surface roughness of the test samples. Field-emission SEM is carried out using a JEOL JSM-7600F (Joel Ltd, Tokyo, Japan) equipped with electron gun operated in the voltage range of 15–25 kV in high vacuum mode. The samples were analysed in backscattering mode only. Platinum coating of 10 nm thickness is applied to the silicone samples by using sputtering technique to avoid artefacts due to charging of the surfaces. Different locations of sample surface are probed to ensure the repeatability of results. Images at 100×, 400× and 1000× magnification were obtained for both molded and 3D printed samples.

#### 2.3.3. Surface Profilometry

The Taylor Hobson Precision tactile stylus instrument (Taylor Hobson Ltd, Leicester, United Kingdom), with a micro-roughness filtering ratio of 2.5 µm in roughness parameter and 0.8 mm Gaussian filter, is used to measure the surface roughness of the silicone implants. The stylus profiler detects changes in surface topography through mechanical contact as it traverses the roughness of the surface. The vertical motion of the stylus is converted to electrical signals by a transducer, which represents the surface profile Z(X) or area topography image Z(X, Y).

#### 2.3.4. Phosphate Buffer Saline (PBS) Absorption Test

The hydroscopic property of silicone meniscus implant was evaluated using a PBS absorption test. Samples of each material were placed in drying oven with constant temperature of 50 °C and dried to constant weight of m_0_, (approximately 30 mm diameter and thickness of 13 mm for standard samples and 4 cm × 2 cm × 1 cm for meniscus implants) were soaked in PBS at temperature of 20 °C, after which they were blotted to remove excess moisture and weighed on a scale m(t). Each silicone sample was weighed after a 24 h soak duration and after a one-week soak duration. In this experiment, the percentage of the weight change was used to characterize the water absorption characteristics. The moisture absorption c(t) can be calculated as follows, c(t) = [m(t) − m_0_]/m_0_ × 100% where m_0_ represents initial weight of the dried sample and m(t) represents the weight of sample measured during the PBS absorption process [35].

#### 2.3.5. X-ray Photoelectron Spectroscopy (XPS)

XPS is used to accurately quantify the elemental composition by spectroscopy of emitted photo electrons with an estimated scanning depth of <10 nm. In this present study, XPS was used to analyze chemical degradation on the surface of the sample. The XPS instrument was of SPECS Om GmbH with magnesium anode operated at an ultra-high vacuum of ~10–9 mbar. The X-ray gun was operated at 10 kV, 300 W. The reference of binding energy was taken as that of the carbon C1s-line (284.6 eV).

#### 2.3.6. Fourier Transform Infrared (FTIR) Spectroscopy

FTIR is used to detect molecular bonding up to a depth of 10 μm by scanning the sample over infrared (IR) frequency range of 400 cm^−1^ to 4000 cm^−1^. In the present study, FTIR was carried out using Shimadzu IR Prestige-21 model equipped with the MIRacleTM single reflection horizontal attenuated total reflection (ATR) accessory having a diamond-zinc-selenium crystal. Before sample measurement, a background scan was performed without any sample to ensure that percentage transmittance obtained at 1000 cm^−1^ was above 25%. For each sample, the diamond crystal was cleaned before measurement to avoid detecting traces from previous samples.

#### 2.3.7. Thermo-Gravimetric Analysis (TGA), Differential Scanning Calorimetry (DSC), and Differential Thermal Gravimetric Analysis (DTG)

TGA was used to investigate the thermal stability of the silicone implant by inducing chemical and physical changes in the sample while performing corresponding measurements of weight change versus temperature. TGA analysis is very useful in determining phase transition temperature during decomposition of the sample.

The following protocol was employed in this study. The results were evaluated using the STAR software. The printed or casted silicone implant end product was used for all measurement. A 10 mg sample of the cured silicone meniscus implant was heated from 25 °C to 550 °C at a heating rate of 30 °C/min in a 30 µL alumina crucible with a closed lid using a purge nitrogen gas flow rate of 80 mL/min. An isothermal state of 550 °C was maintained for 15 min with a continuous nitrogen gas flow of 80 mL/min. Subsequently the sample was heated from 550 to 800 °C at a heating rate of 20 °C/min. An isothermal state of 800 °C was maintained for 10 min with a continuous nitrogen gas flow of 80 mL/min. Finally, the sample was heated from 800 to 950 °C at a heating rate of 20 °C/min with the purge gas switched from nitrogen to air.

TGA was carried out from room temperature to 700 °C using TA Instruments Thermo-Gravimetric Analyzer Q50 model (TA Instruments, Tokyo, Japan) at a constant heat rate of 10 °C/min. DSC analysis was used to explore heat capacity changes during phase transitions. Further study of phase transition was performed using METTLER-TOLEDO DSC1 Model (Mettler-Toledo (S) Pte Ltd., Singapore, Singapore) at the same heat rate of 10 °C/min.

#### 2.3.8. Cell Viability/Cytotoxicity Assays

A fibroblast cell line, L929, was cultured in alpha-minimum essential medium (α-MEM) (Invitrogen, Waltham, MA, USA) supplemented with 9% *v*/*v* heat-inactivated fetal bovine serum (Hyclone, South Logan, UT, USA) and 1% penicillin-streptomycin (Invitrogen, Waltham, MA, USA). Cells were kept in humidified incubator with 5% CO_2_ at 37 °C and were passaged at 80–90% confluence.

Ecoflex silicone were directly printed or casted into a 24-well plate. The silicone samples were washed and soaked in 70/30% ethanol/water mixture for 1 h followed by 1 h of ultraviolet (UV-C) irradiation. The implants were then quickly rinsed with medium containing 1% penicillin-streptomycin before cell seeding at 50,000 cells/well and incubated under culture conditions for 24, 72, and 120 h. At the 24, 72, and 120 h incubation time points, the used culture medium in each well together with any unattached cells was collected and centrifuged at 800 rpm for 5 min. The supernatant was removed, and the cell pellet resuspended in 500 µL of fresh culture medium and seeded back into the same well on the same Ecoflex silicone substrate.

Cell proliferation was investigated with the WST-8 cell proliferation assay (Sigma-aldrich, St. Louis, MO, USA). 50 µL of WST-8 solution was added to 500 µL of cells suspended in culture medium and incubated for 4 h at 37 °C and 5% CO_2_. 50 µL of the supernatant was aliquoted for measurement. The absorbance was measured at 450 nm with a microplate reader (Molecular Devices, San Jose, CA, USA). Cell viability was investigated with the LIVE/DEAD^®^ Viability/Cytotoxicity Kit (Life Technologies, Waltham, MA, USA). The live/dead staining solution was prepared by dissolving 1 µL of 4 mM calcein AM and 4 µL of 2 mM ethidium homodimer-1 in 1 mL of DPBS. The resulting solution was added to each well in 1:1 volume ratio and incubated for 30 min at 37 °C and 5% CO_2_. The stained cells were observed with a fluorescence microscope (Olympus, Tokyo, Japan).

#### 2.3.9. Mechanical Compression

The test was performed according to the ASTM D575 Test Method A using a Shimadzu compression machine (Shimadzu Corporation, Kyoto, Japan). The top compression plate was connected to a load cell (10 kN) and a displacement transducer to record the compression force and displacement, respsectively. Data acquisition of load and crosshead position was undertaken with Horizon Testing Software (Tinius Olsen, Horsham, PA, USA) at constant crosshead displacement rates of 12, 120, 360, 720, and 1000 mm/min. Hence, the sample could expand freely in the transverse direction under compression. The samples were compressed up to 70% strain and engineering stress and strain were measured. The setup is shown in Supplementary Materials Figure S1. The reason for selecting the range of 12–1000 mm/min strain rate was to mimic the human knee’s movement. The slow strain rate mimics static or slow movement e.g., walking and high strain rate mimics fast movement e.g., running.

The stress was derived from the ratio of compression force to average cross-sectional area of the sample. The strain was derived from the ratio of compressive displacement to sample thickness. All tests were repeated three times for each Ecoflex elastomer. All recorded values are mean values with standard errors.

#### 2.3.10. Monotonic Compressive Test Up to Failure

The sample was compressed from 0% strain until complete failure or 70% strain whichever occurs first. The strain level was chosen such that the femoral bone component does not come into contact with the tibial component. Assuming that the stress-strain behavior was linear at the small strain range, the elastic modulus for 30% and 70% strain before failure could be calculated using Hooke’s law, by taking the slope of respective stress-strain curve, as follows E = σ/ε where E is the modulus, σ is engineering stress, and ε is engineering strain.

**Figure 4:**
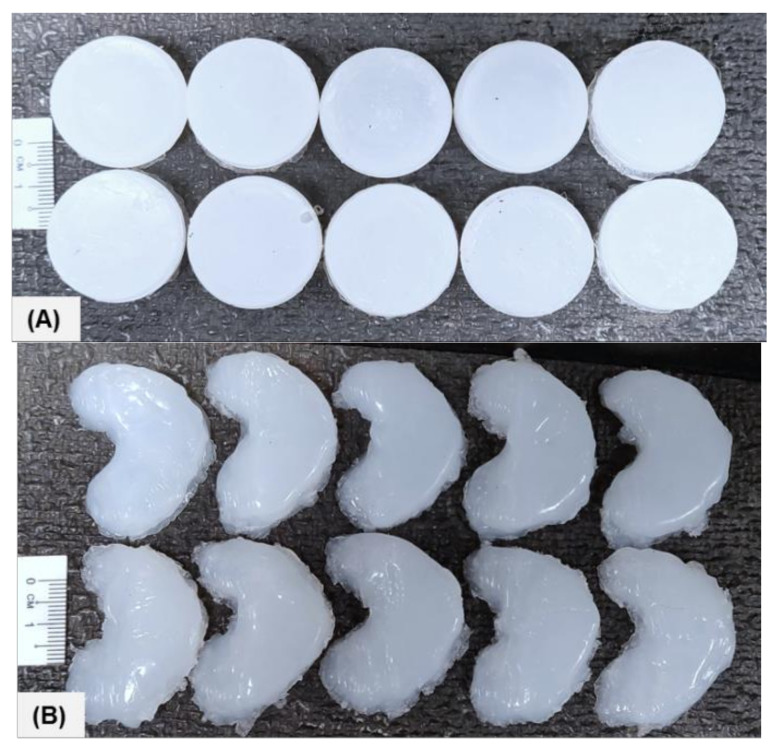
Photograph of the standard and meniscus samples: (**A**) STD-Eco30 samples (top row) and STD-Eco50 samples (bottom row) and (**B**) 3DP-Eco30 samples (top row) and 3DP-Eco50 samples (bottom row).

Low viscosity (Ecoflex-30) and high viscosity (Ecoflex-50) silicone were used to fabricate the silicone meniscus implants for mechanical characterization. Firstly, standard samples were fabricated as per ASTM D575 in order to obtain the compressive modulus of host materials. Thereafter, meniscus samples were 3D printed. The photograph of both standard (STD) and 3D printed (3DP) fully cured samples is shown in Figure 4.

#### 2.3.11. Cyclic Compressive Testing

The sample was compressed from 0% strain until 70% strain and then relaxed back to 0% strain for 1, 4 and 1000 cycles. The maximum number of 1000 cycles was selected to represent the number of steps an average sedentary person would take in a day. Four cycles are chosen for polymer chain stabilization and to investigate Mullin’s effect and hysteresis. The change in mechanical properties of Ecoflex resins versus the number of cycles was observed and compared for both Ecoflex 30 and Ecoflex 50, under different strain rates.

## 3. Results and Discussion

The first part of this section discusses the results of surface characterization of the silicone implants via light microscopy, SEM, PBS absorption tests and surface profilometry. The second part discusses the results of XPS, FTIR, DSC and TGA examining the atomic and chemical composition of the silicone implants. The final section examines the functional results, in terms of the biocompatibility and mechanical properties of the silicone implants.

### 3.1. Light Microscope

**Figure 5:**
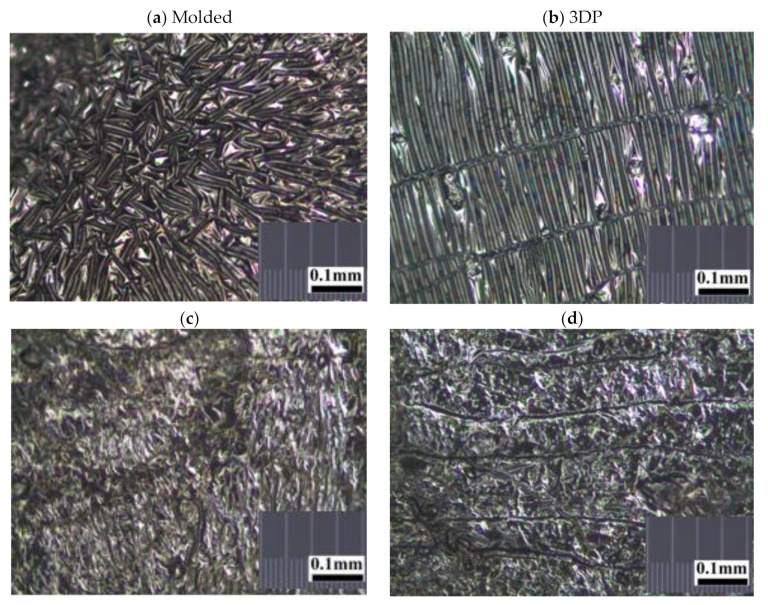
Representative stereomicroscopic images of the core surfaces of silicone meniscus implants. (**a**) surface of molded implant, (**b**) surface of 3D printed implant, (**c**) cross-section of molded implant and (**d**) cross-section of 3D printed implant (20× magnification).

The random, stellated arrangement of the molded silicone samples in surface view (Figure 5a) and in cross-sectional view (Figure 5c) of the molded silicone samples contrast greatly with the regular, striated, laminated layer-by-layer fibrial appearances of the 3DP silicone samples in surface views (Figure 5b) and cross-sectional views (Figure 5d). No obvious manufacturing defect, tears or color discoloration was observed. This difference in architectural arrangement has great implications for mechanical and functional properties of silicone implants and will be discussed in later sections.

### 3.2. Scanning Electron Microscopy

**Figure 6:**
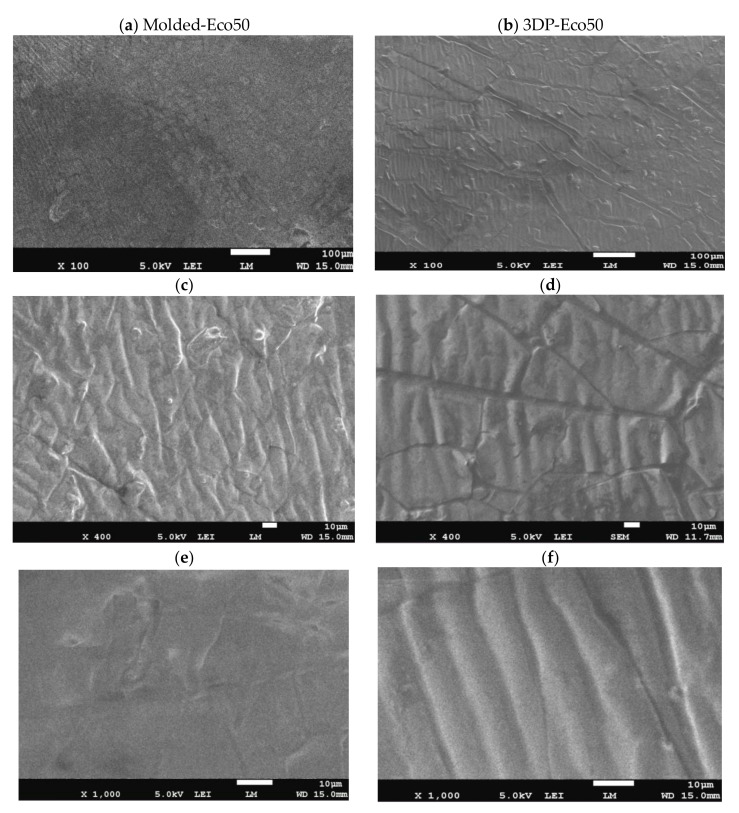
Representative scanning electron microscope (SEM) pictures (magnification 100×, 400× and 1000×) showing the different surface patterns of (**a**,**c**,**e**) molded silicone and (**b**,**d**,**f**) 3D-printed silicone.

Images from SEM clearly demonstrated that different manufacturing methods led to distinct surface patterns of the silicone meniscus. Figure 6a,c,e depicted the irregular, smooth, and diffuse surface patterns on molded silicone implants, whereas Figure 6b,d,f portrayed the wavy, regular, mosaic surface patterns on 3D printed silicone implants. In both groups, SEM images did not reveal any fracture, tear, or heavy metal contamination.

### 3.3. PBS Absorption Test

**Figure 7:**
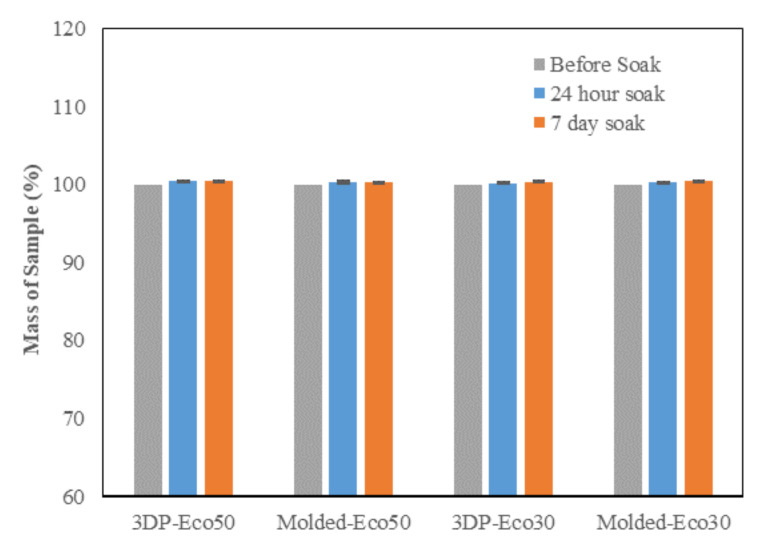
Phosphate buffer absorption test for silicone meniscus implants Ecoflex 50 and 30.

The PBS absorption tests indicated that the water content of both Eco30 and Eco50 silicone meniscus did not show any significant change at pre-soak, 24 h post-soak and 120 h of post-soak (Figure 7). These results are in accordance with those of Bo Gong’s study [35] which proved that water absorption by silicone rubber material obeyed Fick’s law. As shown in Equation (1) below, Fick’s law expresses moisture content c(t) as a function of the diffusion coefficient D and the maximum moisture content c_s_ in wt% at the absorption equilibrium. Based on the results, the maximum moisture content Cs was determined to be 0.36% for Eco30 meniscus implants and 0.47% for Eco50 meniscus implants. These values were slightly higher than the Cs value 0.23% in Bo Gong’s study which assumed that diffusion only occurs from the top and bottom surfaces of their silicone samples. There is no statistically significant difference in water content pass the 24 h time point which clearly indicated that the water content have already stabilised within 24 h of the soaking period. Extrapolating from these results, the meniscus implant should also have attained its final size and shape within 24 h of arthroscopic insertion into the knee joint.

(1) $$c\left(t\right)=c_{s}-c_{s}\frac{8}{\pi^{2}}\overset{20}{\underset{k=1}{\sum }}\frac{1}{{\left(2k-1\right)}^{2}}e^{\left[-\frac{{\left(2k-1\right)}^{2}D\pi^{2}t}{d}\right]}$$

### 3.4. Surface Profilometry

**Figure 8:**
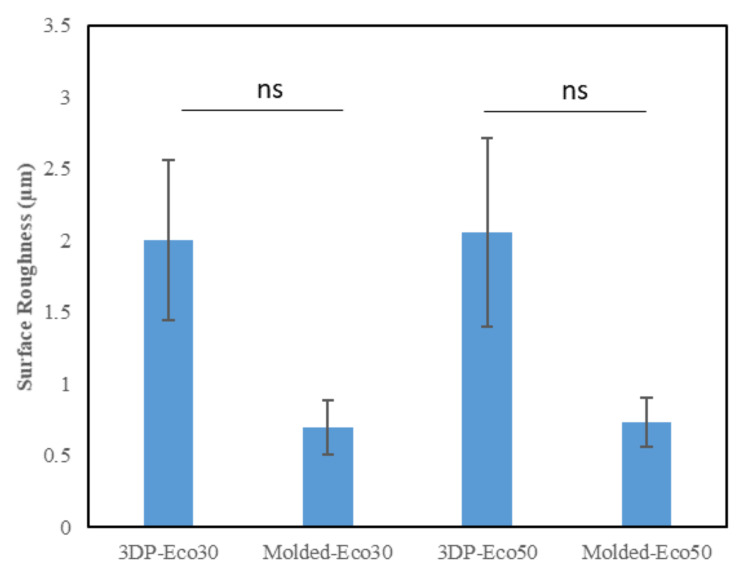
Surface roughness (µm) of the two silicone elastomers (Ecoflex-30 and Ecoflex-50) manufactured by molding and 3D printing.

This method uses a high-resolution probe to detect the surface topography in order to produce a quantitative profile of the surface. The surface of the anterior horn, body and posterior horns of the silicone meniscus were scanned in triplicates. The results from Figure 8 indicated that there is no statistically significant difference in surface roughness between both molded and 3D printed Eco30 and Eco50 silicone implants. However, this is likely due to limitations from the small sample size.

### 3.5. X-ray Photoelectron Spectroscopy

The atomic composition and XPS spectra of molded and 3D printed silicone implant is shown below in Table 2 and in Supplementary Materials Figure S2, respectively. Both molded and 3D printed Eco50 silicone implants display similar silicone composition of 99.7% and 99.8%, respectively (element 14 Si, K alpha-1.740, K beta-1.829, Kab-1.84). The sulphur, iron and copper elements detected are quite possibly additives within the resins. No carbon-black fillers and heavy metal contamination was observed in both groups.

### 3.6. FTIR

**Figure 9:**
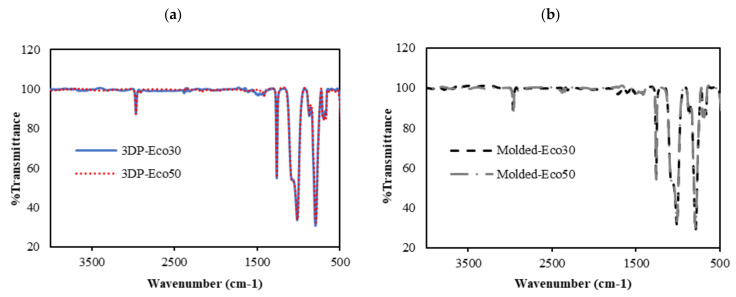
FTIR absorbance spectra of (**a**) 3D printed silicone implant and (**b**) molded silicone sample.

FTIR was used to evaluate changes in molecular structure and functional groups of 3D printed and molded silicone samples. The FTIR results are depicted in Figure 9. The absorption peaks at 3000 cm^−1^ correspond to the stretching vibration of methyl (-CH_3_). The weak absorption peak at 1400 cm^−1^ correspond to the asymmetric Si-CH3 stretching. The stronger absorption peaks at 1255 cm^−1^, 1000–1100 cm^−1^, and 800 cm^−1^ are attributed to Si-CH_3_, Si-O-Si, and Si-(CH_3_)_2_ bonds, respsectively. All these peaks represent characteristic peaks of silicone rubber.

From Figure 9a,b, it can be observed that the FTIR spectra of both 3DP and molded Eco30 and Eco50 silicone samples show exactly the same peaks, indicating that the heat-curing processes (<120 °C) during 3D printing did not alter the chemical composition of the Ecoflex silicone. Also, the flow of silicone resins through the 3D printer did not cause any unwanted contamination of the resins.

### 3.7. DSC/TGA/DTG

TGA measures sample mass as a function of temperature or time and is frequently used in quality control or product development to identify the different components of elastomers such as moisture, solvents, polymers, plasticizers, carbon black or inorganic fillers.

**Figure 10:**
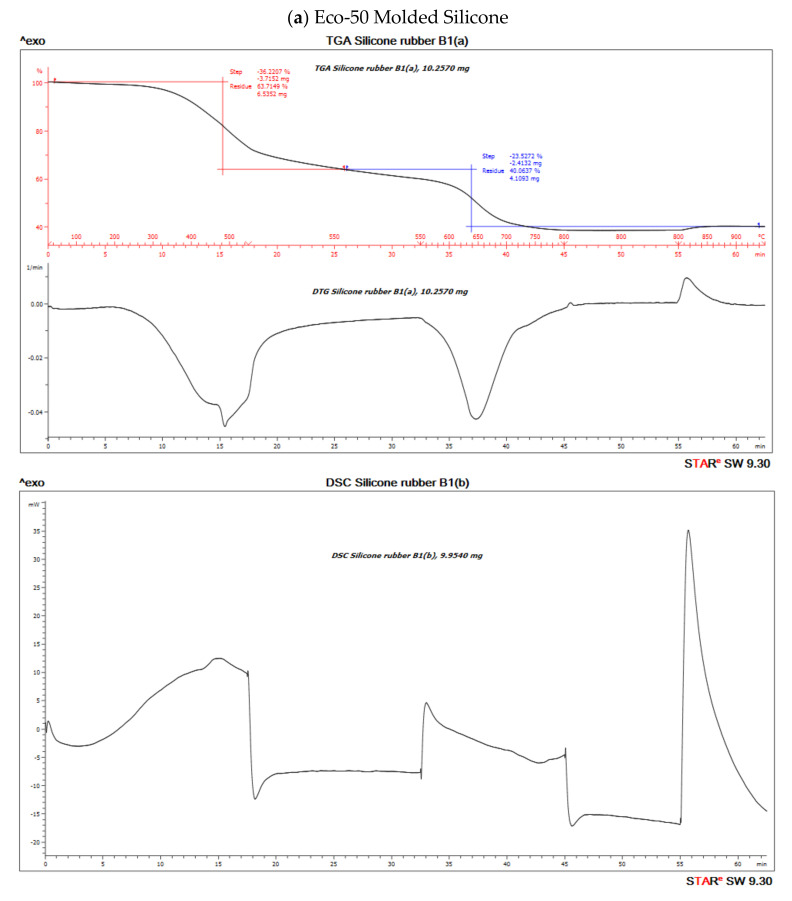

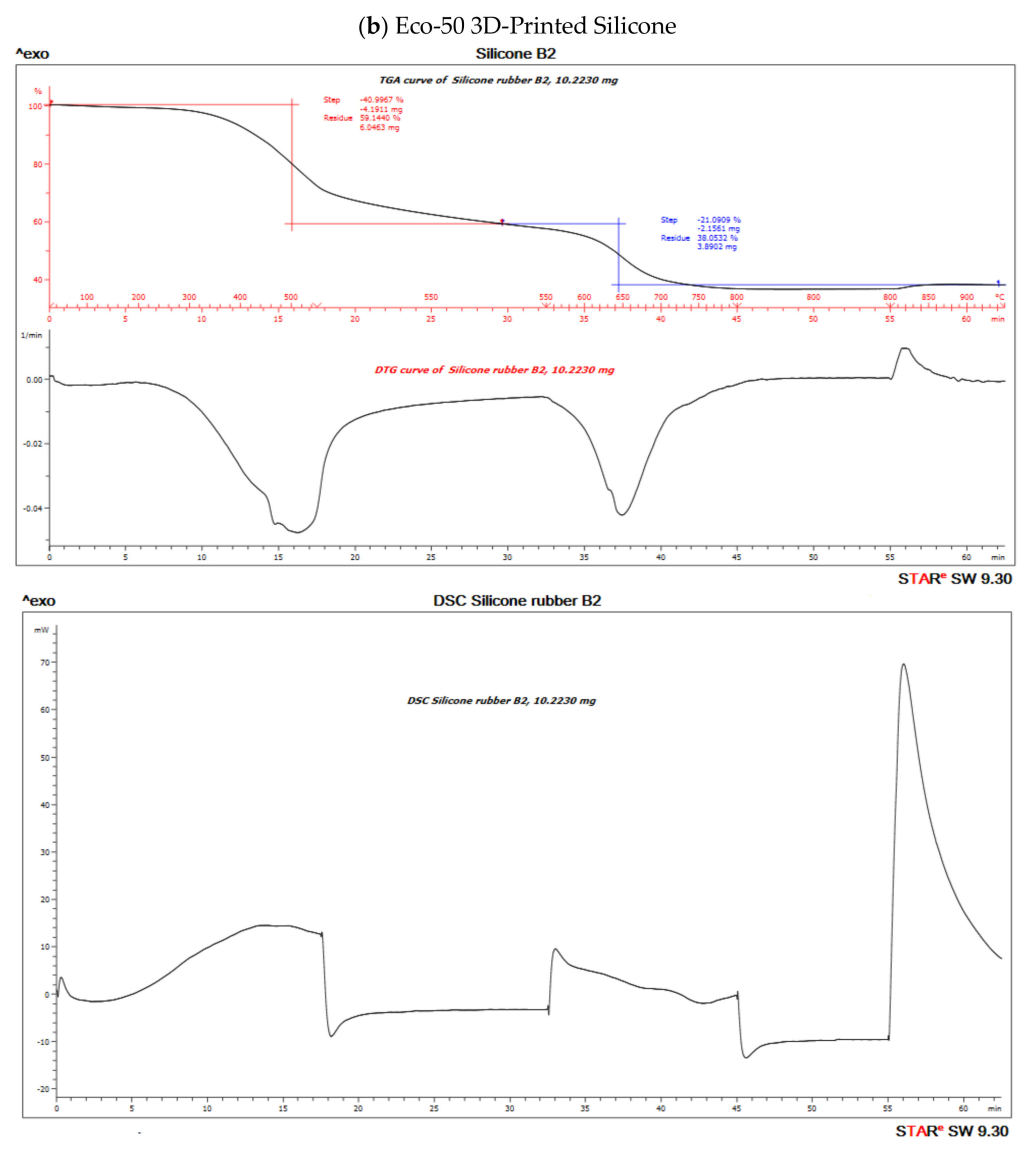
Thermo-gravimetric analysis/differential scanning calorimentry (TGA/DSC) curves of (**a**) molded and (**b**) 3D-Printed silicone Ecoflex 50 measured from 30 to 700 °C at a heat rate of 20 K/min. The TGA curve (red) measures the loss of mass and the DSC curve (black) provides information about endothermic and exothermic effects.

The TGA analysis of Ecoflex silicone elastomer comprises of three steps (Figure 10). The DTG curve (the first derivative of the TGA curve) is used to determine the temperature limits for evaluation. The first step below ~300 °C amounts to 3.1% and corresponds to the loss of small quantities of relatively volatile un-crosslinked chemicals. Between 300 and 550 °C, pyrolysis of the silicone takes place. Polymer content was determined to be about 59.937% and 61.95% in the molded and 3D printed silicone sample, respectively (Table 3). The residual silica content was determined to be about 40.073% in the molded sample and 38.053% in the 3D printed sample. To oxidize the carbon black formed during pyrolysis, the atmosphere is switched from nitrogen to air at 600 °C. With many elastomers, the amount of carbon black formed during pyrolysis can be neglected. The carbon black filler content can therefore be determined from the third step between 600 and 700 °C and yields a value of 40.03% and 38.05% in molded and 3D printed silicone samples, respectively. The final residue comprised of inorganic fillers such as silicates of oxides. The first derivative of the TGA curve is represented by the DTG curve and can be used to quantify the decomposition rate. 

Both molded and 3D printed methods of Eco50 silicon samples show similar percentages of thermal degradation of silicone (Supplementary Materials Figure S3). At any heating temperatures, more heat inflow is required for Eco50 samples to attain the same temperature, when compared to Eco30 samples, as shown in Supplementary Materials Figure S3a. The results also prove that 3D printed silicone samples will remain thermally stable for heat sterilization processes, such as autoclaving at 121 °C, exhibiting no significant loss of mass for temperatures up to 300 °C.

### 3.8. Cytocompatibility of Molded and 3D-Printed Silicone

Implantation of biomaterials could cause host reactions such as blood-material interactions, tissue injury, foreign body reaction, inflammation, and fibrosis/fibrous capsule development. In the very early process of implantation, blood/material interactions occur with protein adsorption to the biomaterial surface and development of a blood-based transient provisional matrix that forms on and around the biomaterial.

**Figure 11:**
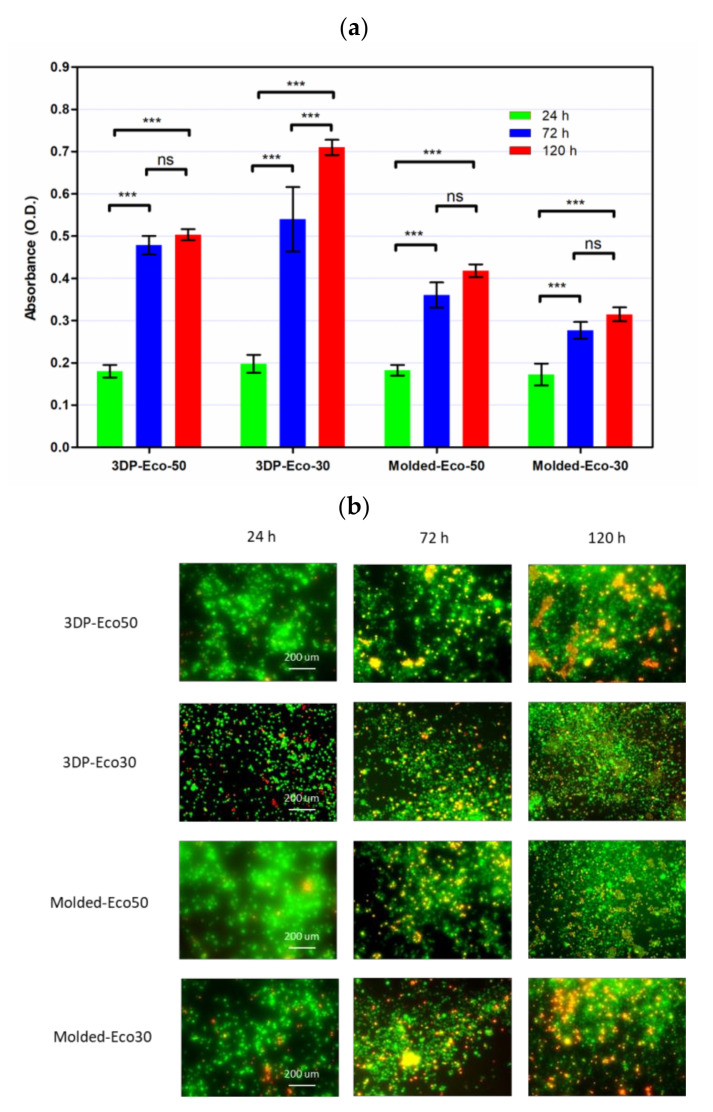
(**a**) Cell proliferation of re-seeded L929 cells on printed/casted Eco50/Eco30 substrates after 24, 72, and 120 h culture was quantified based on the WST-8 cell proliferation assay. (**b**) Fluorescent images of re-seeded L929 cells on printed/casted Eco50/Eco30 substrates after 24, 72, and 120 h culture. Cells were stained with the Live/Dead^®^ cell viability assay. Statistical significance between groups was assessed using two-way analysis of variance (ANOVA) followed by Bonferroni post-tests. ns = *p* > 0.05 and *** = *p* < 0.001.

In order to investigate the extent of foreign body reaction, L929 fibroblasts were seeded and allowed to proliferate in culture medium on molded or 3D-printed silicone samples for 24, 72, and 120 h. The silicone samples were already subject to sterilization with ethanol and UV-C irradiation before use in our studies and did not exhibit any significant changes in properties. After washing steps, the cells were re-seeded to study cell adhesion to silicone samples. The results from Figure 11b demonstrated that cells remained spherical and did not form an extended morphology typical of attached cells in a 2D culture (Supplementary Materials Figure S4). The results indicate that fibrosis/fibrous capsule development does not take place even when fibroblasts were in direct contact with silicone surface up to 120 h. However, the re-seeded fibroblasts continued to show contact-inhibited growth for up to 120 h and started to form cell clusters on the surface due to the low cytotoxicity of the silicone samples (Figure 11a). Furthermore, it appears that a correlation exists between surface roughness and contact-inhibited growth of cells on silicone surface. This could be attributed to more cells physically anchoring themselves to the surface of 3D printed silicone by virtue of the increased surface roughness as shown in Figure 8.

### 3.9. Mechanical Compression Test

The overall non-linear stress-strain responses of both Ecoflex30 and Ecoflex50 silicone elastomers under monotonic compressive loading up to failure showed linear elastic region at low strain level and followed by a non-linear region at higher strain level before failure.

**Figure 12:**
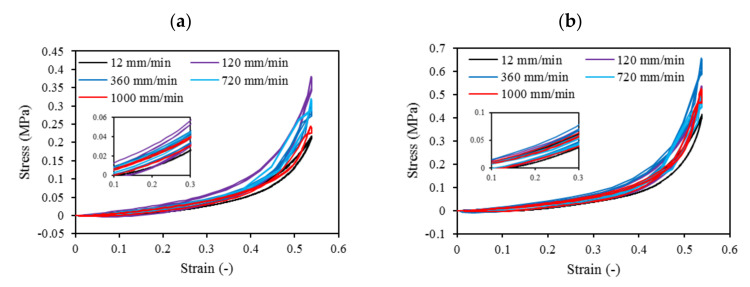
Four-cycle cyclic stress-strain for (**a**) STD-Eco30 and (**b**) STD-Eco50 at strain rates of 12, 120, 360, 720, and 1000 mm/min.

Cyclic loading at 4-cycles for Eco30 and Eco50 silicones are shown in Figure 12. The graphs also showed hysteresis and strain hardening, which are viscoelastic properties of silicone. The initial compressive modulus at the linear region is the functioning modulus of the meniscus implant. It can be estimated from the slope of the curve in the 10% to 30% strain range. In hysteresis, the loop area enclosed by the loading and unloading pathways represents the energy loss in the system. This area is more pronounced at higher strain rates and with Eco50 silicone resins, indicating more mechanical damping in the system due to higher viscosity. However, the hysteresis effect decreases with an increasing number of cycles of cyclic loading.

Mechanical properties of soft polymers in cyclic loading is one of the important material properties to be understood for their successful implementation in the actual system for the long run. Usually, Ecoflex of higher Shore hardness exhibits greater stiffness and larger hysteresis. The viscoelastic behavior of Ecoflex silicone elastomers has been extensively investigated by Liao et al. [41,42]. In their experiments, tensile properties and hysteresis of Ecoflex elastomers with different hardness have been reported. Our cyclic test results in a compression mode are highly coherent to Liao et al. [41,42] cyclic test results in a tensile mode.

Strain hardening is the strengthening of material properties with repeated elastic deformation. This phenomenon is observed in both Eco30 and Eco50 silicone implants. Strain hardening is observed especially at low strain rates up to 200 mm/min before stabilizing at higher strain rates at 800 mm/min. A less viscous sample takes more cycles for strain hardening effect to stabilize [34].

**Figure 13:**
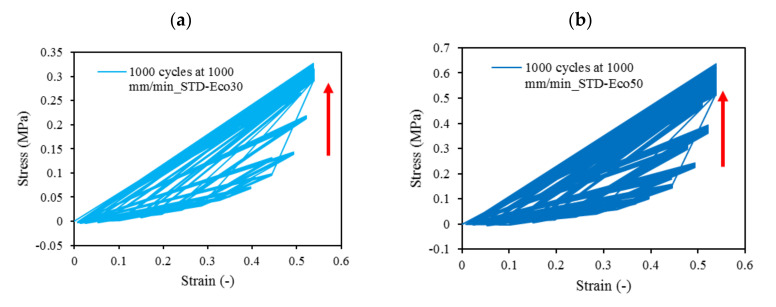
1000-cycle cyclic stress-strain for (**a**) STD-Eco30 and (**b**) STD-Eco50, at a strain rate of 1000 mm/min.

**Figure 14:**
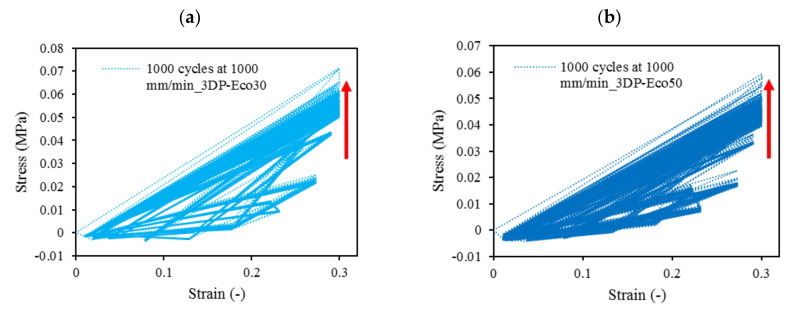
The 1000-cycle cyclic stress-strain for (**a**) 3DP-Eco30 and (**b**) 3DP-Eco50, at a strain rate of 1000 mm/min.

**Figure 15:**
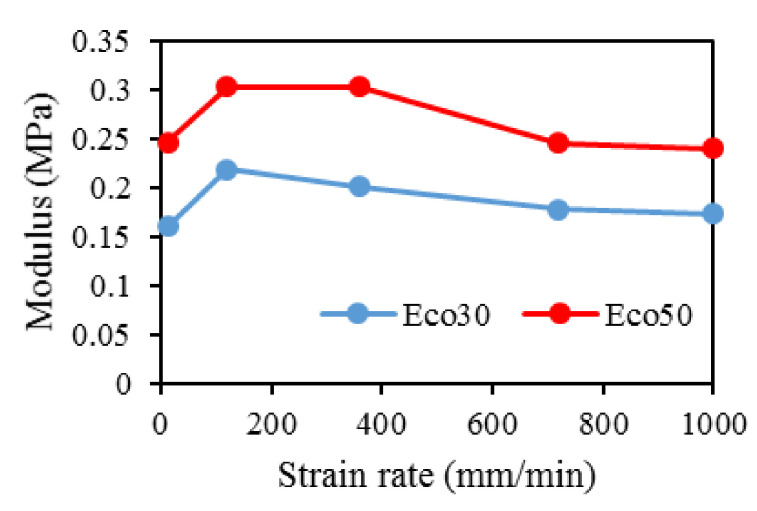
Modulus for Eco30 and Eco50 at different strain rates.

At all values of strain rates, Eco50 silicone implants consistently demonstrated higher compressive modulus and stress values than their Eco30 counterparts. At cyclic loading of 1000-cycles, Eco50 meniscus implants also demonstrated higher stress at failures, when compared to their Eco30 meniscus implant counterparts (Figure 13 and Figure 14). In Figure 12, the red line represents the 4-cycle run at a strain rate of 1000 mm/min and is almost a straight line when tested to a strain of 0.3, after which a curvilinear line is observed up to a strain of 0.5. Lines of other colors represent 4-cycle run at other strain rates. Figure 13 and Figure 14 showed 1000 overlapping lines after a run of 1000-cycle at 1000 mm/min. In the first four cycle runs, similar trends were observed as in Figure 12. Subsequent lines with steeper gradients showed strain hardening characteristics. Comparing Figure 12, Figure 13 and Figure 14 at the same strain rate of 1000 mm/min, similar trends were observed, ie linear up to a strain of 0.3 and curvilinear from strain of 0.3 to 0.5. The modulus of Eco30 and Eco50 samples are 0.161, 0.219, 0.202, 0.179, and 0.174 MPa and 0.247, 0.304, 0.304, 0.246, and 0.241 MPa, respectively, at a strain rate of 12, 120, 360, 720, and 1000 mm/min (Figure 15).

## 4. Conclusions

This study concludes that the silicone 3D printing process does not permanently change the overall physical, biochemical, or mechanical properties of the 3D printed silicone meniscus. There was no statistically significant difference in surface roughness properties and water absorption characteristics between molded and 3D Printed samples of Eco30 and Eco50. There is no shelf life for these 3D printed models. Results from FTIR and TGA/DSC demonstrated the chemical and thermal stabilities of Ecoflex silicone rubber, respectively. Both Eco30 and Eco50 demonstrated viscoelastic properties of strain hardening and hysteresis. At all strain rates, Eco50 silicone meniscus implants and standard samples consistently displayed higher stiffness and modulus as compared to Eco30 samples. Finally, cytotoxicity tests proved that both Eco30 and Eco50 silicone implants are biocompatible.

## Figures and Tables

**Table 1:** Ecoflex sample technical data [40].

	Mixed Viscosity (cps)	Specific Gravity (g/cc)	Specific Vol. (cu. in./lb.)	Pot Life (min)	Cure Time (h)	Shore Hardness	Tensile Strength (psi)	100% Modulus (psi)	Elongation at Break (%)	Die B Tear Strength (pli)	Shrinkage (in./in.)
Ecoflex 50	8000	1.07	25.9	18	3	00–50	315	12	980	50	<0.001
Ecoflex 30	3000	1.07	26.0	45	4	00–30	200	10	900	38	<0.001

**Table 2:** Atomic Composition obtained for molded and 3D Printed Silicone Implant.

Samples	Si (%)	S (%)	Fe (%)	Cu (%)
Eco50 molded implant	99.739	0.253	0.005	0.003
Eco50 3D-printed implant	99.816	0.174	0.005	0.01

**Table 3:** Percentage (%) polymer content of material and % residual silicates/oxides.

Samples	% Polymer Content	% Residual Silicates/Oxides
Eco-50 Molded Silicone implant	59.937	40.073
Eco-50 3D Printed Silicone implant	61.95	38.053

## Supplementary Materials

The following are available online at https://www.mdpi.com/2073-4360/12/9/2136/s1: Figure S1. Compression plates set-up used for static compression test. Figure S2. XPS spectra of (a) molded and (b) 3D printed silicone implant. Figure S3. Combined (a) DSC, (b) TGA, and (c) DTG graphs of 3D Printed and Molded Eco30 and Eco50 samples. Figure S4. Fluorescent and corresponding optical images of L929 cells attached and proliferating on surfaces of 24-well cell culture plates after 24, 72, and 120 h culture. Cells were stained with the Live/Dead® cell viability assay.
