# Supplementary material for: 3D Printed Silicone Meniscus Implants: Influence of the 3D Printing Process on Properties of Silicone Implants
Source: Polymers (Basel). 2020 Sep 18;12(9):2136. doi: 10.3390/polym12092136 (PMC7570003; doi:10.3390/polym12092136)
Supplement: Supplementary file 1 [file polymers-12-02136-s001.pdf]

Supporting Information

**3D Printed Silicone Meniscus Implants: Influence of the 3D Printing Process on Properties of Silicone Implants**

*Eric Luis[1], Houwen Matthew Pan[2], Anil Bastola[1], Ram Bajpai[3], Swee Leong Sing[1], Juha Song[2], and Wai Yee Yeong[1,] **

[1]Singapore Centre for 3D Printing, School of Mechanical and Aerospace Engineering, Nanyang Technological University, 50 Nanyang Drive, Singapore 639798
Email: wyyeong@ntu.edu.sg

[2]School of Chemical and Biomedical Engineering, Nanyang Technological University, 70 Nanyang Drive, Singapore 639798

[3]Center for Population Health Sciences, Lee Kong Chien School of Medicine, Nanyang Technological University, 11 Mandalay Road, Singapore 308232

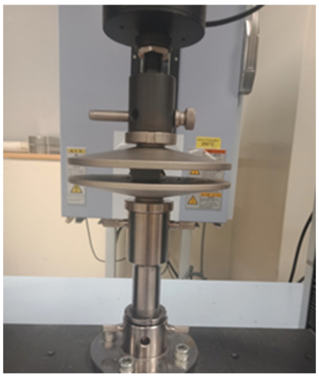

**Figure S1**. Compression plates set-up used for static compression test.

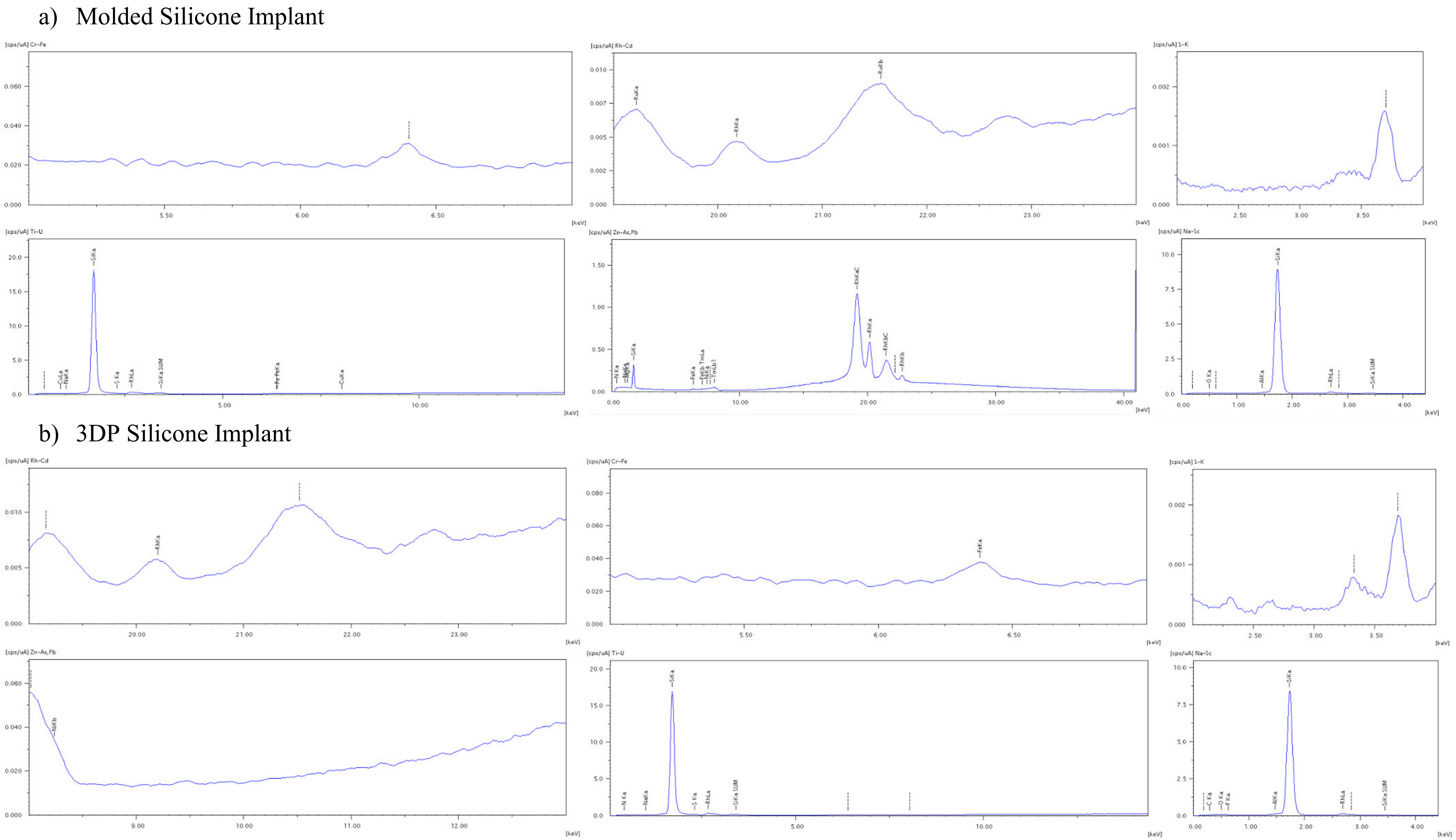


**Figure S2.** XPS spectra of (a) molded and (b) 3D printed silicone implant.

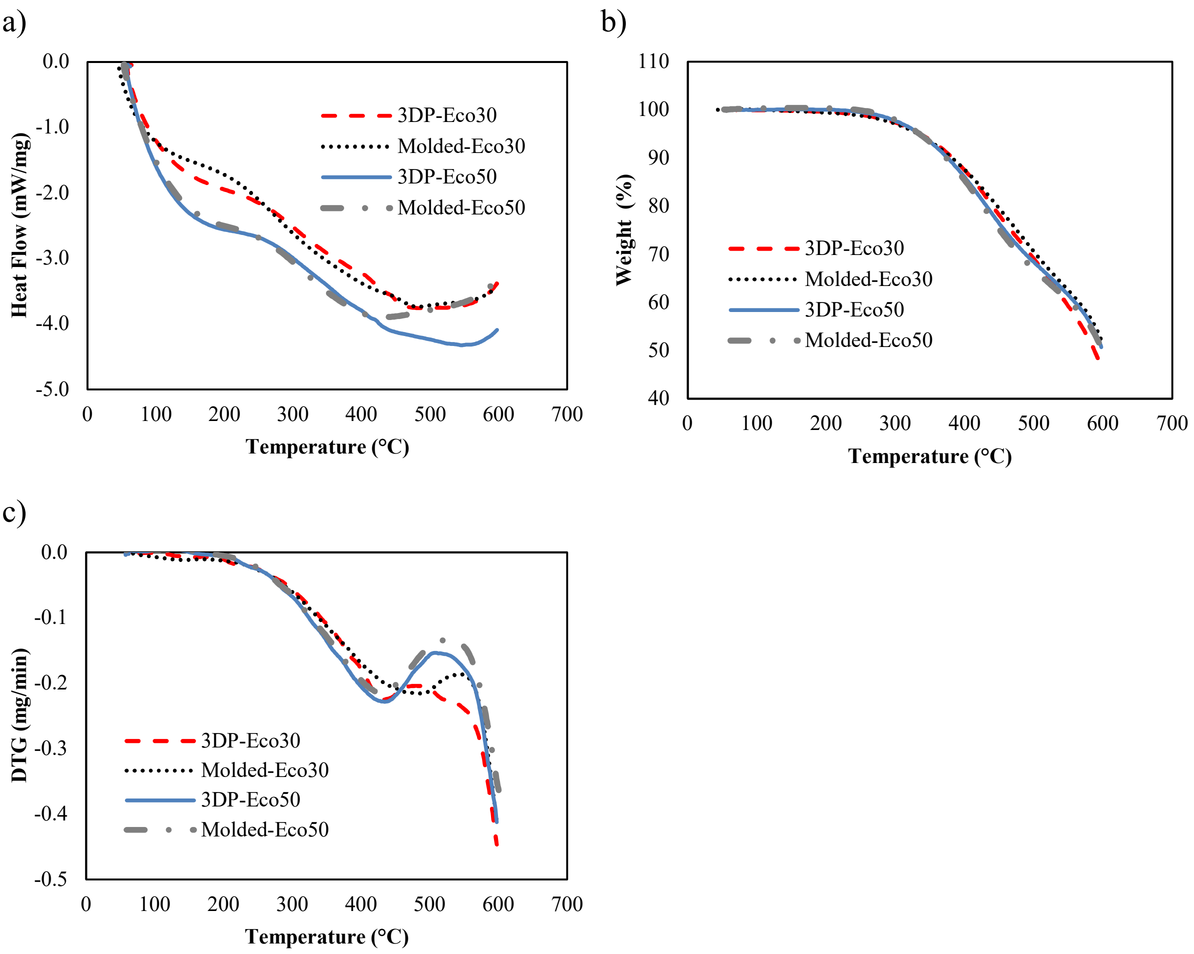


**Figure S3**. Combined (a) DSC, (b) TGA, and (c) DTG graphs of 3D Printed and Molded Eco30 and Eco50 samples.

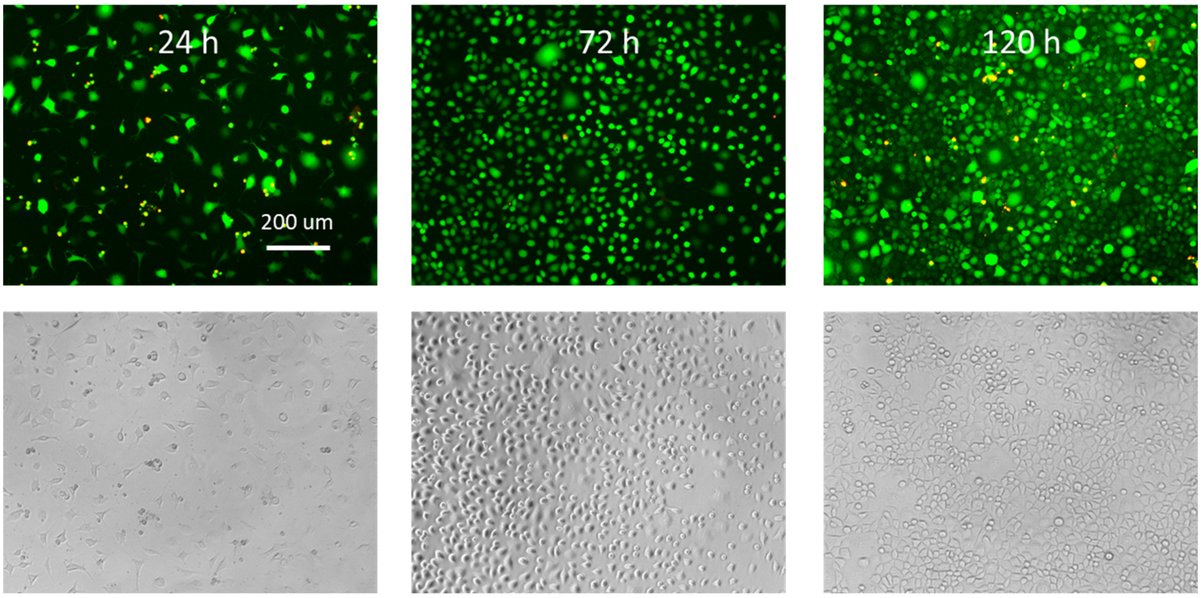


**Figure S4**. Fluorescent and corresponding optical images of L929 cells attached and proliferating on surfaces of 24-well cell culture plates after 24, 72, and 120 h culture. Cells were stained with the Live/Dead® cell viability assay.
